# Identifying and controlling emerging foodborne pathogens: research needs.

**DOI:** 10.3201/eid0304.970416

**Published:** 1997

**Authors:** R L Buchanan

**Affiliations:** USDA ARS Eastern Regional Research Center, Wyndmoor, PA 19038, USA. rbuchanan@arserrc.gov

## Abstract

Systems for managing the risks associated with foodborne pathogens are based on detailed knowledge of the microorganisms and the foods with which they are associated--known hazards. An emerging pathogen, however, is an unknown hazard; therefore, to control it, key data must be acquired to convert the pathogen from an unknown to a known hazard. The types of information required are similar despite the identity of the new agent. The key to rapid control is rapid mobilization of research capabilities targeted at addressing critical knowledge gaps. In addition, longer-term research is needed to improve our ability to respond quickly to new microbial threats and help us become more proactive at anticipating and preventing emergence. The type of contingency planning used by the military in anticipating new threats serves as a useful framework for planning for new emergence.


### Identifying and Controlling Emerging Foodborne Pathogens: Research Needs

**Robert L. Buchanan**
USDA ARS Eastern Regional Research Center, Wyndmoor, PA, USA

Systems for managing the risks associated with foodborne pathogens are based on detailed knowledge of the microorganisms and the foods with which they are associated—known hazards. An emerging pathogen, however, is an unknown hazard; therefore, to control it, key data must be acquired to convert the pathogen from an unknown to a known hazard. The types of information required are similar despite the identity of the new agent. The key to rapid control is rapid mobilization of research capabilities targeted at addressing critical knowledge gaps. In addition, longer-term research is needed to improve our ability to respond quickly to new microbial threats and help us become more proactive at anticipating and preventing emergence. The type of contingency planning used by the military in anticipating new threats serves as a useful framework for planning for new emergence.

The microbiologic safety of food has been advanced substantially by the introduction and implementation of Hazard Analysis and Critical Control Points (HACCP). HACCP provides a systematic conceptual framework for identifying hazards and focusing efforts on the proper functioning of key food production, processing, and marketing steps. When applied appropriately, HACCP is a cost-effective means of controlling known hazards in foods. Its successful implementation depends on knowledge of such issues as the pathogenic microorganisms' virulence, cultural characteristics, ways in which they contaminate the food, effects of food processing and preparation on their survival, and food consumption patterns. Because it requires substantial knowledge, HACCP cannot be expected to control unknown hazards, such as emerging foodborne pathogens. Therefore, controlling a new foodborne microbial threat requires moving the hazard as quickly as possible from being unknown to being known. The key to this transition is the timely acquisition of needed research data. This article identifies classes of research information needed and discusses a conceptual approach for addressing unknown microbial threats.

Address for correspondence: Robert L. Buchanan, USDA ARS Eastern Regional Research Center, 600 East Mermaid Lane, Wyndmoor, PA, 19038, USA; fax: 215-233-6445; e-mail: rbuchanan@arserrc.gov.

### Anticipating the Next Emerging Pathogen

Two types of emergence are encountered with pathogenic foodborne microorganisms. A true emergence, where a microorganism that had not been identified as a public health threat begins to cause disease, is relatively rare. More common is reemergence, where a known microorganism causes disease in a new way, for example, by causing new types of infections, being associated with new foods, or appearing in new geographic locations. For both types, the operational requirement is to control an unanticipated public health threat. The timeliness of response is critical since the public health and economic costs of an emerging pathogen are directly related to the time between its emergence and its control.

The events that lead to emergence are often complex, with the cause often being obscure and only indirectly related to the new agent. Past emergence of foodborne pathogens has been associated with changes in microbial genotypes, demographics, food production and processing methods, marketing and preparation practices, medical diagnostics, globalization of the food industry, changes in consumer education, and general socioeconomic trends (1-3). Planning for a microbial threat is a challenge because one does not know what the agent will be, what food it will be associated with, or where or when emergence will occur. While there are several potential ways of anticipating and responding to microbial threats, the contingency planning used by the military to anticipate threats seems well suited for emerging pathogens. Military contingency planning can be viewed as having four major components: intelligence, personnel and facilities, rapid response, and strategic planning.

Intelligence is the gathering of medical, scientific, and other information that allows emergence to be identified. In the United States, this role is filled to a great extent by the Centers for Disease Control and Prevention (CDC). In addition to providing information on known foodborne pathogens, CDC works with local public health agencies and the medical and scientific communities to investigate new disease syndromes and identify unrecognized foodborne pathogens. This type of intelligence gathering played a pivotal role in the recent recognition of *Cyclospora* as a cause of foodborne gastroenteritis. CDC's new sentinel site program, FoodNet, is expected to greatly enhance the identification of new foodborne diseases. However, these surveillance activities are largely limited to the United States, whereas an effective intelligence system for foodborne disease must be worldwide. For example, *Cyclospora* was identified as a likely foodborne or waterborne pathogen in Asia and South America before an outbreak was reported in the United States. Intelligence related to foodborne disease can be acquired from several sources: the World Health Organization's surveillance program, the U.S. military's international network of laboratory and medical investigators, medical and scientific reports, and the Internet. The Internet is increasingly an important source of intelligence related to emerging pathogens; through news groups and bulletin boards such as ProMed, scientists and public health practitioners share their experiences on almost a real-time basis. Such advances in intelligence gathering are critical to reducing the time between emergence and control. However, limiting intelligence to medical considerations is not enough: intelligence gathering must include awareness of changes and advances in food production methods, agricultural practices and conditions, veterinary medicine, environmental and water microbiology, food technologies, consumer trends, and general socioeconomic conditions.

The second component of contingency planning is ensuring sufficient personnel and facilities to characterize a new biologic agent and develop control strategies. The inability to predict the agent or the associated food, coupled with the degree of specialization required of investigators, requires a broad range of capabilities and resources. However, no one organization is likely to maintain the capabilities needed to deal with all contingencies. If we were to follow the military pattern, we would have reserve groups that could be mobilized as needed. However, even this approach requires planning and support to ensure the needed expertise and facilities. For example, the number of researchers and laboratories studying *Clostridium botulinum* has dropped to a point where it would be difficult to rapidly mobilize a research team, despite this pathogen's history of reemerging in a surprisingly wide range of foods.

Rapid mobilization of resources is the third component. This component is particularly important for free-living infectious agents because one goal is to limit their dissemination to prevent them from establishing secondary reservoirs. It is much easier to fight a small, contained war than a global one. The mobilization of resources to respond to an emergence must be appropriate to the severity of the threat. Overreacting hurts the credibility of the entire system, while underreacting increases both the public health and economic impact. Rapid response efforts have focused at identifying new agents and removing suspect food from the marketplace, two key initial steps. However, research to prevent another occurrence of the emerging pathogen has been much less organized and timely.

The fourth component of contingency planning, strategic planning, is actually the first chronologically. This is the phase where members of war colleges pose "what we would do if" scenarios and plan appropriate responses. This type of contingency planning has generally received attention in relation to emerging pathogens only in connection with the use of biologic warfare agents. This process relies on futurist thinking to consider how changes in society, economics, technology, agriculture, medicine, and international trade may affect the microbiologic safety of the food supply. Such a broad view is needed because more general events or trends in society cause most disease emergence. This type of strategic planning is undertaken with the realization that the probability of any specific "what if" scenario is low, but the probability that one scenario will materialize is extremely high.

### Research Needs

Research, an integral part of responding to a new foodborne microbial threat, is the key for moving a new or reemerging biologic agent from being an unknown pathogen to being one for which control measures are available. Two areas of research can be classified on the basis of time constraints. Acute research needs are deficiencies in knowledge that must be addressed to establish control of an emerging pathogen. This research is highly targeted and specific for the microorganism and food of concern; it must be accomplished as quickly as possible. Acute needs generally require applied research, although basic research may have to be conducted if the deficiencies in knowledge are great. The second class encompasses longer-term basic and applied research needs not mandatory to immediate control.

### Acute Needs

While the data needed for any single emerging biologic agent are highly specific, acute research needs fall into general categories that are virtually the same for all new pathogens. Common research questions include the following: Are methods available for detecting and categorizing the agent? What food is the vehicle for the pathogen? How do the implicated foods become contaminated? What is the pathogen's reservoir in nature? Is the pathogen's presence in contaminated food the result of an error or breakdown in normal controls? Does the pathogen grow in foods? Does the pathogen survive normal food processing, distribution, and preparation? How infectious/toxigenic is the pathogen? Are there subpopulations of consumers at increased risk for this pathogen? Is the pathogen's ability to cause disease restricted to specific strains with identifiable virulence characteristics? Answering these questions requires specific data that do not differ substantially from pathogen to pathogen (Table).

Table. Research data needed for most emerging foodborne pathogens

| Research area | Knowledge gaps |
|---|---|
| Detection methods | Sampling and enrichment techniques<br>Cultivating<br>Biochemical/taxonomic char.<br>Antibodies for capture and differentiation<br>Subtyping<br>Virulence-associated char.<br>Detecting injured or viable-but-nonculturable cells |
| Microbial ecology | Contaminated foods<br>Reservoirs and routes of transmission<br>Life cycles<br>Geogr. range and seasonality<br>Route of contamination and location of pathogen in food |
| Pathogenicity | Dis. char. and diagnosis<br>Sequelae<br>Host range<br>Infectious dose<br>Subpopulations at risk<br>Animal models |
| Growth characteristics | Free-living vs. obligate parasite<br>Growth requirements<br>Temperature<br>pH<br>Water activity<br>Oxygen |
| Survival characteristics | Heat resistance<br>D-values<br>Z-values<br>Susceptibility to anti-microbial food additives<br>Acid resistance<br>Sensitivity to disinfectants or dessication<br>Sensitivity to radiation<br>UV<br>Ionizing |
| Control | Effectiveness of food preservation<br>Inspection systems to segregate contaminated materials |

The criteria for classifying needs as acute are reasonably straightforward: Is the research needed to prevent a recurrence of the disease or to modify current HACCP plans? However, these questions have different priorities, which depend on when the information is needed. To deal with emerging pathogens, we should learn from modern business practices, especially the concept of "just-in-time" research. Little consideration has been given to how to assess and set research priorities for emerging foodborne pathogens. One attempt was provided as an appendix of the U.S. Pathogen Reduction Task Force. A relatively simple decision tree used a series of questions to identify what research was the limiting step in responding to the foodborne pathogen (4).

The timeliness of addressing research needs must be an integral part of the planning process, but has been generally overlooked. Past research mobilization efforts for new foodborne microbial threats can be best described as haphazard, likely because they reflect the way research is funded. The traditional means of ensuring strong research programs, competitive funding of projects after proposals have undergone extensive peer review, is time consuming and often not appropriate for the acute phase of responding to an emerging foodborne pathogen. Further, the peer-review process tends not to select the often mundane research needed during the acute phase of an emergence. Two alternative approaches may be more effective. The first is to have a series of designated laboratories that have as part of their mission and funding the task of being able to modify their research programs to address acute research needs. Such laboratories would need to have a critical mass of facilities and expertise in various aspects of food safety microbiology. The second approach is to have a group of reserve scientists with unique expertise or access to facilities not available at the designated laboratories or needed to supplement those capabilities. Funds could be earmarked to noncompetitively fund such reserve scientists on an as-needed basis, with the understanding that research needs designated as acute would take precedence over other research needs.

### Longer-Term Needs

The three areas of longer-term research associated with emerging pathogens are amenable to more traditional funding. The first area, specific to the new pathogen, consists of research for improvements or alternatives to the detection and control methods initially devised. With initial disease control established, basic and applied research can seek to understand the microorganism and develop more optimal approaches for its prevention, control, or elimination. The second area concerns activities to help reduce the time between the emergence of a pathogen and its initial control (e.g., improved surveillance through the development of new diagnostic methods and further identification and characterization of virulence determinants and modes of pathogenicity to accelerate detection of new agents). Just as important as acquiring research data is rapid data dissemination. The continuing development of computer-based information networks is a component of this second research area.

The third area focuses on identifying research factors that will allow new microbial threats to be anticipated. Of necessity, the current response to emerging pathogens is almost entirely reactive. The public health community detects a new syndrome, and only then is research mobilized, often during a crisis. While reactive response will always be part of dealing with emerging microbial threats, a more proactive approach is needed if prevention is to be even partially realized. In military terms, war is the last resort and represents the failure of diplomats to predict and prevent a crisis. Microbial threats, like wars, do not spontaneously emerge but are the result of a series of events or conditions. There is a need to reexamine how food is produced, processed, marketed, and prepared to identify conditions that contribute to emergence. For example, organic acids are used extensively throughout the food industry to control spoilage and pathogenic microorganisms. Archer (5) hypothesized that over time, exposure to pH conditions that stress but do not kill may lead to the emergence of hardier and possibly more virulent foodborne pathogens. It is already well established that the induction of acid tolerance can enhance both the survival and virulence of foodborne pathogens (6). Further, one of the basic tenets of microbial genetics is that conditions that kill most, but not all, of a bacterial population foster the development of resistance. This is supported by recent studies that suggest that bacterial stress responses may select for hypermutability (7,8). While these findings do not mean that organic acids should not be used as a tool for controlling foodborne pathogens, they suggest that proactive research should be conducted to find ways of using these agents that minimize the potential for resistance. Proactive research, including research that might appear unrelated to the emergence of foodborne pathogens, can draw on the already substantial body of basic research related to the conditions and requirements for gene transfer among biologic agents. For example, Baur et al. (9) reported on the conditions that led to the competence of *Escherichia coli* for genetic transformation in freshwater environments.

Maximal competence occurred when the bacterium was exposed to 2 mM $Ca^{2+}$ as temperatures increased from 10°C to 20°C. With such information, researchers could examine food processing operations to determine the presence and importance of such conditions. For example, fruits and vegetables are often treated with calcium under fluctuating temperatures to enhance the texture during later processing.

A key to being more proactive in addressing the threat of microbial foodborne pathogens—consideration of root causes—will likely require food microbiologists to become involved in nontraditional research areas. If new biologic agents arise as the result of changes in technology, society, or global economics, predicting and preventing emergence will ultimately require better understanding of how such factors influence pathogen introduction and dissemination.

### Conclusions

One of the critical lessons of the past 10 years is that we cannot become complacent about infectious diseases (1). Only a few diseases (e.g., smallpox) have actually been eliminated. The rest, including virtually all foodborne diseases, we hold in check, winning battles but not the war. Eventually, our weapons (e.g., antibiotics) become obsolete; pathogens (e.g., *E. coli)* become more dangerous; or we become complacent. Contingency planning must be developed and undertaken with a long-term commitment. Without that commitment and without understanding that planning is successful when problems are avoided or minimized, programs of this type lapse quickly. In the long term, the costs of planning, both in terms of economics and human suffering, are a fraction of those incurred as the result of the emergence of a major microbial threat.

### References

1. Lederberg J, Shope RE, Oaks S. Emerging infections, microbial threats to health in the United States. Washington (DC): U.S. National Academy of Sciences Press; 1992.
2. Nathanson N. The emergence of infectious diseases: societal causes and consequences. American Society of Microbiology News 1996;62:83-8.
3. Potter ME. Factors for the emergence of foodborne disease. In: Amgar A, editor. Food Safety '96: Proceedings of the 4th ASEPT International Conference. Laval, France: ASEPT; 1996:185-95.
4. United States Department of Agriculture. Subcommittee report for the pathogen reduction taskforce: identifying research and education needs. Washington (DC): USDA Food Safety and Inspection Service; 1994.
5. Archer DL. Preservation microbiology and safety: evidence that stress enhances virulence and triggers adaptive mutations. Trends in Food Science Technology 1996;7:91-5.
6. Foster JW. Low pH adaptation and the acid tolerance response of *Salmonella typhimurium*. Crit Rev Microbiol 1995;21:215-37.
7. Moxon ER, Rainey PB, Nowak MA, Lenski RE. Adaptive evolution of highly mutable loci in pathogenic bacteria. Curr Biol 1994;4:24-33.
8. LeClerc JE, Li B, Payne WL, Cebula TA. High mutation frequencies among *Escherichia coli* and *Salmonella* pathogens. Science 1996;274:1208-11.
9. Baur B, Hanselmann K, Schlimme W, Jenni B. Genetic transformation in freshwater: *Escherichia coli* is able to develop natural competence. Appl Environ Microbiol 1996;62:3673-8.
